# From Nature to Treatment: The Impact of Pterostilbene on Mitigating Retinal Ischemia–Reperfusion Damage by Reducing Oxidative Stress, Inflammation, and Apoptosis

**DOI:** 10.3390/life14091148

**Published:** 2024-09-11

**Authors:** Beáta Pelles-Taskó, Réka Szekeres, Barbara Takács, Anna Szilágyi, Dóra Ujvárosy, Mariann Bombicz, Dániel Priksz, Balázs Varga, Rudolf Gesztelyi, Zoltán Szabó, Zoltán Szilvássy, Béla Juhász

**Affiliations:** 1 Department of Pharmacology and Pharmacotherapy, Faculty of Medicine, University of Debrecen, Nagyerdei St. 98., H-4032 Debrecen, Hungary; pelles-tasko.beata@med.unideb.hu (B.P.-T.); szekeres.reka@med.unideb.hu (R.S.); takacs.barbara@pharm.unideb.hu (B.T.); dr.szilagyi.anna@med.unideb.hu (A.S.); bombicz.mariann@pharm.unideb.hu (M.B.); priksz.daniel@pharm.unideb.hu (D.P.); varga.balazs@pharm.unideb.hu (B.V.); gesztelyi.rudolf@pharm.unideb.hu (R.G.); szilvassy.zoltan@med.unideb.hu (Z.S.); 2 Department of Emergency Medicine, University of Debrecen Clinical Centre, Nagyerdei St. 98., H-4032 Debrecen, Hungary; ujvarosy.dora@med.unideb.hu (D.U.); szabo.zoltan@med.unideb.hu (Z.S.)

**Keywords:** retinal ischemia–reperfusion, pterostilbene, electroretinography (ERG), GFAP, HSP70, PARP1, NFκB, neuroprotective, reactive oxygen species (ROS), protective effects

## Abstract

Retinal ischemia–reperfusion (I/R) injury is a critical pathogenic mechanism in various eye diseases, and an effective therapeutic strategy remains unresolved. Natural derivatives have recently reemerged; therefore, in our present study, we examined the potential therapeutic effects of a stilbenoid that is chemically related to resveratrol. Pterostilbene, recognized for its anti-inflammatory, anti-carcinogenic, anti-diabetic, and neuroprotective properties, counteracts oxidative stress during I/R injury through various mechanisms. This study explored pterostilbene as a retinoprotective agent. Male Sprague Dawley rats underwent retinal I/R injury and one-week reperfusion and were treated with either vehicle or pterostilbene. After this functional electroretinographical (ERG) measurement, Western blot and histological analyses were performed. Pterostilbene treatment significantly improved retinal function, as evidenced by increased b-wave amplitude on ERG. Histological studies showed reduced retinal thinning and preserved the retinal structure in the pterostilbene-treated groups. Moreover, Western blot analysis revealed a decreased expression of glial fibrillary acidic protein (GFAP) and heat shock protein 70 (HSP70), indicating reduced glial activation and cellular stress. Additionally, the expression of pro-apoptotic and inflammatory markers, poly(ADP-ribose) polymerase 1 (PARP1) and nuclear factor kappa B (NFκB) was significantly reduced in the pterostilbene-treated group. These findings suggest that pterostilbene offers protective effects on the retina by diminishing oxidative stress, inflammation, and apoptosis, thus preserving retinal function and structure following I/R injury. This study underscores pterostilbene’s potential as a neuroprotective therapeutic agent for treating retinal ischemic injury and related disorders.

## 1. Introduction

Ischemia–reperfusion (I/R) injury refers to the tissue damage that occurs when blood supply to a specific area is initially restricted or cut off (ischemia) and then restored (reperfusion). While reperfusion is essential to restore oxygen and nutrients to the deprived tissues, this process paradoxically leads to additional injury. The sudden influx of oxygen during reperfusion generates reactive oxygen species (ROS) and triggers inflammatory responses, which can result in cellular dysfunction, apoptosis, necrosis, and overall tissue and organ damage. Ischemia–reperfusion injury is a fundamental mechanism underlying various clinical conditions, including heart attacks, strokes, and even retinal neurodegenerative disorders. This pathological feature is present in age-related macular degeneration (AMD), diabetic retinopathy (DR), central retinal artery occlusion (CRAO), and glaucoma [1,2,3], which are the most common causes of total blindness due to degeneration and demise of retinal ganglion cells [4,5]. Nonetheless, neuroinflammation and oxidative stress have been identified as pivotal contributors to its development [6]. During the ischemic phase, characterized by restricted or blocked blood flow to the retina, a reduction in oxygen availability leads to metabolic dysregulation and cellular stress. This cascade of events culminates in the generation of reactive oxygen species (ROS) as metabolic byproducts, initiating various processes such as mitochondrial dysfunction, the activation of NADPH oxidase, and inflammatory responses leading to oxidative stress [7]. The dysregulated redox state perpetuates further cellular injury, exacerbating tissue damage and functional impairment [8]. Following retinal ischemia, the restoration of blood flow, known as reperfusion, leads to a surge of oxygen and nutrients, which can cause several deleterious changes in the retinal tissue, a phenomenon commonly referred to as ischemia–reperfusion (I/R) injury. During reperfusion, reactive oxygen species (ROS) are generated. The abrupt influx of oxygen results in ROS overproduction, which causes oxidative damage to various cellular components, including lipids, proteins, and DNA. Due to its high metabolic demand and rich content of polyunsaturated fatty acids, the retina is particularly susceptible to oxidative stress. ROS attack membrane lipids, leading to lipid peroxidation, which compromises membrane integrity, alters permeability, and can result in cell death.

Excessive ROS production also damages mitochondria, impairing their function, which disrupts ATP production essential for cell survival and exacerbates oxidative stress. Reperfusion triggers the activation of microglia, the immune cells of the retina. Activated microglia release pro-inflammatory cytokines, such as TNF-α and IL-1β, contributing to further tissue damage. Additionally, reperfusion increases the expression of adhesion molecules on retinal endothelial cells, leading to leukocyte infiltration. These leukocytes release additional inflammatory mediators, exacerbating inflammation and tissue injury. The combined effects of oxidative stress, mitochondrial dysfunction, and inflammation can lead to apoptosis, particularly in retinal ganglion cells (RGCs) and photoreceptors, characterized by the activation of specific cell death pathways that result in controlled cell disassembly [1,9,10,11].

Various studies have investigated the factors influencing I/R injury. Previous findings indicate that heat shock protein 70 (HSP70), functioning as a molecular chaperone protein, and glial fibrillary acidic protein (GFAP), an intermediate filament protein, are upregulated as part of the cellular stress response during I/R injury. Additionally, levels of NFκB, a transcription factor, were found to increase in response to diverse stimuli such as oxidative stress and inflammatory cytokines in the context of I/R injury. Accumulating evidence from multiple studies suggests that poly(ADP-ribose) polymerase 1 (PARP1), an enzyme critical for DNA repair and genomic stability, plays a significant role in retinal ischemia–reperfusion injury by contributing to oxidative stress, inflammation, and neuronal apoptosis [12,13,14,15,16].

Currently, there is no definitive cure for retinal injury caused by I/R, other than the standard treatment of restoring blood flow to save under-perfused tissues. In recent years, natural compounds with potent antioxidant and anti-inflammatory properties have emerged as promising therapeutic candidates for managing retinal ischemic injury [17,18,19]. Pterostilbene (3′,5′-dimethoxy-resveratrol), a stilbenoid structurally akin to resveratrol, is abundant in blueberries, grapes, and various plants [20,21,22]. Blueberry extract, often included in eye vitamins, is prized for its rich content of antioxidants, particularly anthocyanins and flavonoids [23]. It is characterized by a chemical structure comprising two phenolic rings connected by a methylene bridge, which confers a spectrum of pharmacological effects, including antioxidative, anti-inflammatory, neuroprotective, and vasoprotective actions [24,25]. Pterostilbene operates through multifaceted mechanisms to counteract oxidative stress, activating antioxidant enzymes like superoxide dismutase and catalase to neutralize harmful reactive oxygen species [26]. Moreover, pterostilbene suppresses the expression of pro-inflammatory cytokines, thereby dampening the inflammatory cascade provoked by ischemic insult [27,28]. Additionally, it exerts a protective influence against apoptosis, a critical mediator of apoptotic cell death, and attenuates mitochondrial dysfunction-associated apoptosis pathways [29]. Furthermore, pterostilbene has exhibited neuroprotective effects in diverse experimental models of neurological disorders [30], suggesting its potential extension to the retina, where it may safeguard retinal function and prevent neuronal cell death following ischemia–reperfusion injury [28,31,32].

Our study aimed to investigate the impact of pterostilbene on both healthy eyes and eyes subjected to I/R-induced injury. We sought to determine whether pterostilbene can inhibit retinal ganglion cell (RGC) apoptosis, retinal gliosis, and inflammation, which are critical events in retinal degeneration following ischemia–reperfusion injury.

## 2. Materials and Methods

### 2.1. Animals and Groups

We employed male adult Sprague Dawley (SD) rats, aged 18 weeks and weighing between 500 and 600 g, obtained from Charles River Laboratories International, Inc. (Wilmington, MA, USA). These rats were kept under standard conditions, with temperatures maintained between 22 and 24 °C, at the Department of Pharmacology and Pharmacotherapy, University of Debrecen, Hungary. A 12 h light–dark cycle was applied, and the rats could access water and standard rat chow freely. Prior to the commencement of the study, a two-week preliminary adaptation phase was provided. After acclimatization, the rats were randomly allocated into two groups (*n* = 10/group): one receiving vehicle (mucilage) treatment and the other receiving pterostilbene treatment. Mucilage was made from a viscosity enhancer, hydroxyethylcellulose, and water according to a Hungarian standard recipe published as an official preparation formula in Formulae Normales VIII—a Collection of Standard Recipe Samples. It is a colorless or slightly yellowish, transparent, almost odorless, tasteless viscous fluid. It mixes with water in all proportions. It was purchased from the University Pharmacy. Pterostilbene (source of pterostilbene: Apollo Scientific, product code: BIP1700, purity: ≥97% (HPLC), form: white solid, synonyms: Pterocarpus marsupium, 3′,5′-dimethoxy-resveratrol, 3,5-dimethoxy-4′-hydroxystilbene, 4-[(1E)-2-(3,5-dimethoxyphenyl)ethenyl]phenol, empirical formula: C_16_H_16_O_3_) was administered at a dosage of 5 mg/kg/day [33], once daily, for a duration of five weeks. The substances were administered through a gastric tube to ensure accurate dosing. Animal groups are shown in Table 1. We followed institutional and ethical guidelines to use as few animals as possible, taking into account potential losses. In accordance with animal ethics principle 3R, ischemia–reperfusion injury was specifically induced in the left eye of rats. The unblinded right eyes served as healthy controls. This approach not only reduced the overall number of animals but also helped to normalize any unintended systemic effects of pterostilbene between the treated and untreated groups. Rats were treated ethically and sparingly, and all methods in this study were conducted according to the ‘Principles of Laboratory Animal Care’ outlined in EU Directive 2010/63/EU. The local Ethics Committee of the University of Debrecen approved all experimental protocols (approval number: 14/2022/DEMÁB).

### 2.2. Ocular Ischemia–Reperfusion

General anesthesia was achieved with ketamine–xylazine (100/10 mg/kg), which was followed by the administration of a local anesthetic agent (oxibuprocaine, Humacain 4 mg/mL eye drops, Teva Ltd., Debrecen, Hungary). Subsequently, ischemia was induced in the left eyes of the SD rats using a methodology previously reported [34].

Briefly, bent forceps were used to gently protrude the left eye of each SD rat, and a polyester fiber surgical suture (Mersilene, 2 mm, Ethicon Inc., Cincinnati, OH, USA) was used to create a slip knot behind the eye bulb. To effectively restrict blood supply to the retina for a desired time period, this slip knot was tightened around the blood vessels of the eye. Ischemia induction was verified macroscopically by fundoscopic examination with an ophthalmoscope. During anesthesia, the rats’ eyes were protected from dehydration using a specialized eye gel (Vidisic, Bausch & Lomb, Berlin, Germany). The ischemic state lasted 60 min after which the slip knot was loosened to restore the flow of blood in retinal arteries. The visual confirmation of sufficient reperfusion was performed using ophthalmoscopy.

### 2.3. Electroretinography

Hand-held Multi-species ElectroRetinoGraph, a Ganzfeld-type flash electroretinograph (HMsERG, OcuScience, Henderson, NV, USA), was utilized for both stimulus generation and data acquisition. The ERG measurements adhered to previously described methodologies [35]. The rats, divided into vehicle-treated (*n* = 10) and pterostilbene-treated (*n* = 10) groups, were anesthetized with a mixture of ketamine–xylazine (100/10 mg/kg). Upon achieving deep anesthesia, mydriasis was induced using topical cyclopentolate (Humapent, Teva Ltd., Debrecen, Hungary), following which the animals were dark-adapted for 20 min. During the procedure, the animals were positioned in a prone position on a heated pad (ATC 2000, WPI, Sarasota, FL, USA) to maintain a constant body temperature of 37 °C. Gold-coated corneal contact lens electrodes (ERG-jet Contact Lens Electrode, Fabrinal SA, La Chaux-De-Fonds, Switzerland) were placed on each eye, with reference and ground stainless steel needle electrodes inserted subcutaneously above the jaw and tail base, respectively. Conductive gel was applied to the cornea to ensure optimal electrical contact and hydration throughout the procedure.

ERGs were recorded from both eyes simultaneously using a Ganzfeld dish. Single-flash images were acquired under both dark-adapted (scotopic) and light-adapted (photopic) conditions. The bandpass filter width was set to 1–300 Hz. The single white flash stimulus intensity ranged from −2.5 to 1 log cd·s/m^2^, and light adaptation was performed with a 30 cd·s/m^2^ backlight for 10 min before recording photopic responses. At each flash intensity, 10 responses were averaged, with the interval between stimuli varying between 2 and 20 s depending on the flash intensity. Data analysis was performed using software provided by the ERG system manufacturer. The dark-adapted oscillatory potential (OP) measurements were derived from ERG waveforms recorded for 3000 mcd·s·m^−2^ flash stimuli, with bandwidth filtering of 100–300 Hz after recording. For the measurement of OP amplitudes, the highest positive and the lowest negative peaks were measured from a baseline set to 0 µV. The absolute values of the two numbers were then summed. The implicit time is the time required for the highest positive peak to be formed after the flash. Four individual OP averages were given for each eye. Data analysis was performed using software provided by the ERG system manufacturer (ERGView 4.380, Ocuscience, Henderson, NV, USA).

### 2.4. Western Blot

Immediately following euthanasia, both eyes of the animals were carefully removed from the orbit and promptly immersed in liquid nitrogen for preservation until subsequent molecular biological examinations (*n* = 4 per group). Whole-eye samples were then pulverized and homogenized with a homogenization buffer comprising 25 mM Tris, 25 mM NaCl, 1 mM Na–orthovanadate, 10 mM NaF, 10 mM Na–pyrophosphate, 10 mM okadaic acid, 0.5 mM EDTA, 1 mM PMSF protease inhibitor cocktail, and distilled water, all sourced from Sigma-Aldrich Merck KGaA, Darmstadt, Germany. The resulting solution was further homogenized using a disperser (IKA-WERKE, Staufen, Germany). Following centrifugation at 2000 rpm for 10 min at 4 °C, the cytosolic and mitochondrial proteins were collected with the supernatant, while the pellet containing the nuclear fraction was dissolved and incubated for 1 h in homogenization buffer supplemented with Triton X 100 surfactant (Sigma-Aldrich Merck KGaA, Darmstadt, Germany). Following the initial centrifugation step (14,000 rpm, 10 min, 4 °C), the supernatant containing nuclear proteins was carefully aspirated. Subsequently, the supernatant containing cytosol and mitochondria underwent further centrifugation (10,000 rpm for 20 min at 4 °C), and the resulting supernatant, containing the cytosolic fraction, was collected. The total protein concentration in 10 μL of the supernatants (nuclear and cytoplasmic) was measured using a spectrophotometer (FLUOstar Optima, BMG Labtech, Ortenberg, Germany) and a BCA assay kit (QuantiPro BCA Assay Kit, Sigma-Aldrich Merck KGaA, Darmstadt, Germany). The remaining volume was either mixed with Laemmli sample buffer (Sigma-Aldrich Merck KGaA, Darmstadt, Germany) or stored at −80 °C for further analysis.

Proteins were separated by SDS–polyacrylamide gel electrophoresis (12% gel, 25 mA for approximately 220 min). Subsequently, the blotting of proteins to a nitrocellulose membrane (GE Healthcare, Darmstadt, Germany) was performed at 25 V for 90 min. The membrane was then blocked in a 5% BSA solution (Sigma-Aldrich Merck KGaA, Darmstadt, Germany) and incubated overnight at 4 °C with primary antibodies in TBST solution. The primary antibodies used included anti-histone H3 (detecting histone 3, ~17 kDa), anti-beta-actin (detecting beta-actin, ~42 kDa), anti-HSP70 (~70 kDa), anti-PARP1 (~113 kDa), GFAP (55, 48 kDa), and anti-NFκB (~50 kDa) antibodies. The membranes were then washed with TBST for 3 × 10 min and incubated with secondary antibodies (anti-mouse or anti-rabbit) conjugated with horseradish peroxidase enzyme. The blots were visualized using WesternBright™ enhanced chemiluminescent substrate (Advansta Inc., Menlo Park, CA, USA) and a LiCor C-Digit blot scanner (LI-COR Inc., Lincoln, NE, USA). Scanned images were analyzed using Image Studio Digits software (version 5.2, LI COR Inc., Lincoln, NE, USA), including normalization to the background and standardization to a housekeeping protein (histone H3 or beta-actin). Three Western blots from all treatment groups were analyzed.

### 2.5. Histology

Following euthanasia, the eyes of four animals per group were promptly excised from the orbit, with the upper portion of each eyeball marked for subsequent orientation. Subsequently, the eye bulbs were infused with and immersed in 4 °C paraformaldehyde solution (PFA, pH 7.4, 4% in phosphate buffer containing 10 g paraformaldehyde, 50 µL 10 N NaOH, 25 mL 10× PBS, and 200 mL ddH_2_O) for 24 h to ensure the proper fixation of the retina. The following day, corneas were removed to ensure the thorough removal of PFA, and tissue samples were washed in water for 1 h. The tissue specimens were then preserved in 70% alcohol until further histological processing. Histological processing involved sequential dehydration in 70%, 90%, and 100% ethanol, followed by clearing in xylene and infiltration/embedding in Histowax (Histolab Products AB, Gothenburg, Sweden). Subsequently, the paraffin-embedded eye tissue blocks were sectioned frontally with a microtome into 5 µm thick sections. Sections near the optic disc were selected for further processing. After deparaffinization and the rehydration of the sections, hematoxylin–eosin (H & E) staining was performed as follows: The sections were stained for 1.5 min with hematoxylin (Gill-type, GHS2128, Sigma-Aldrich Merck KGaA, Darmstadt, Germany). Following staining, sections were rinsed in running tap water until they turned blue, followed by a 3 s staining with eosin (Eosin Y, Alcoholic solution, 3801600E, Leica Biosystems Richmond, Inc., Mumbai, Maharashtra, India). Images were captured from the inferior part of the retina, near the optic disc, using a Nikon Eclipse 80i microscope equipped with a DS-Fi3 Microscope Camera, utilizing a 40× objective lens (Nikon Plan Fluor 40×/0.75 DIC M/N2 ∞/0.17 WD 0.66, Nikon Europe B.V., Amstelveen, The Netherlands). Measurements were conducted using Nikon NIS-Elements BR microscope software (Ver5.41.00).


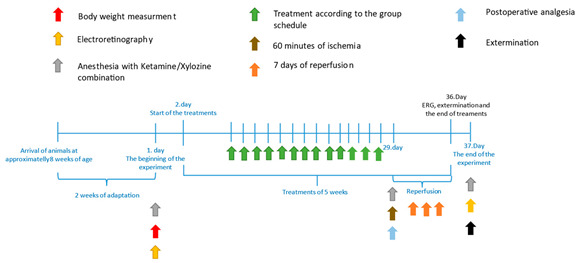


### 2.6. Statistical Analyses

The results are presented as means ± standard error of the mean (SEM). The normality of the data within the groups was assessed using the D’Agostino–Pearson test. Differences in means among the groups were analyzed using a one-way analysis of variance (ANOVA) followed by Tukey’s post hoc test. We applied Mann–Whitney tests for statistical analysis in instances where this method was applicable. A *p*-value less than 0.05 was considered statistically significant. GraphPad Prism software for Windows (version 7.00, GraphPad Software Inc., La Jolla, CA, USA) was used for statistical analysis.

## 3. Results

### 3.1. Electroretinography

**Figure 1:**
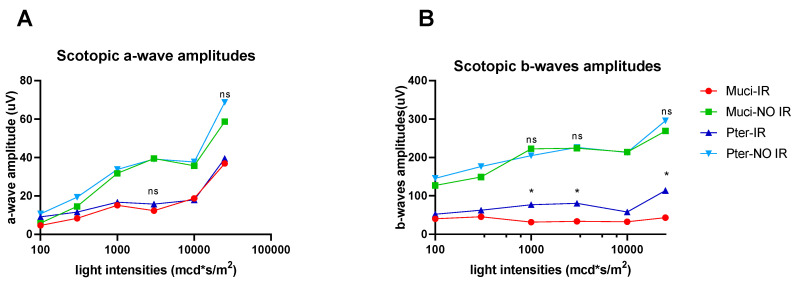
Comparison of a-wave and b-wave responses in ERG: (**A**) amplitude analysis of a-wave responses across different experimental groups; (**B**) amplitude analysis of b-wave responses across different experimental groups. Data represent mean ± SEM; * *p* < 0.05. ns = no significant difference.

The electroretinography (ERG) study revealed notable findings. In both a-wave and b-wave responses, the vehicle-treated ischemic–reperfusion group (MUCI IR) exhibited the lowest amplitudes compared to other experimental groups (Figure 1A,B).

**Figure 2:**
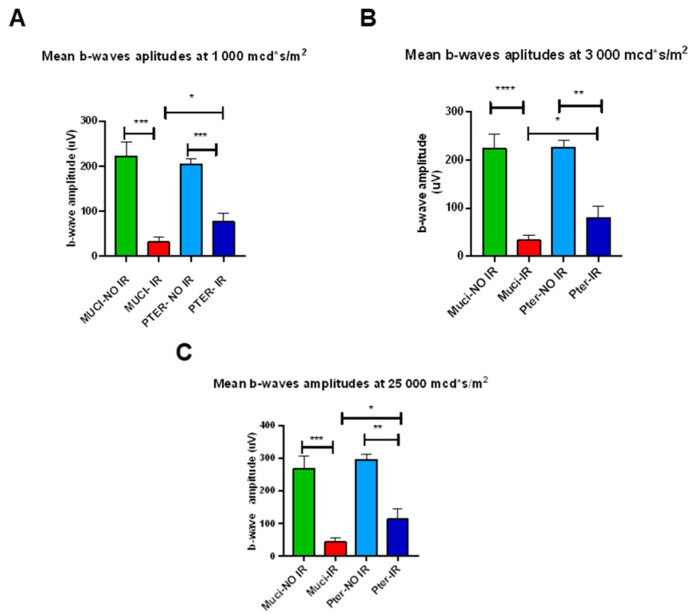
Statistically most significant comparisons in scotopic ERG measurements; flash intensity: mcd·s·m^−2^: (**A**) average b-wave amplitudes (µV) of the different groups at 1000 mcd·s·m^−2^ light intensity; (**B**) average b-wave amplitudes (µV) at 3000 mcd·s·m^−2^ light intensity; (**C**) average b-wave amplitudes (µV) at 25,000 mcd·s·m^−2^ light intensity (µV). All results are plotted as group average ± SEM. Statistically significant comparisons are denoted by * *p* < 0.05; ** *p* < 0.01; *** *p* < 0.001; **** *p* < 0.0001.

Specifically, the mean amplitude of b-waves was significantly diminished in the vehicle-treated ischemic–reperfusion group (MUCI IR) compared to both the vehicle-treated non-ischemic control (MUCI NO IR) and pterostilbene-treated ischemic–reperfusion (PTER IR) groups. Additionally, the mean b-wave amplitudes of the pterostilbene-treated ischemic–reperfusion (PTER IR) group demonstrated higher values than those observed in the Mucilago-treated ischemic–reperfusion (MUCI IR) group (Figure 2A–C). 

**Figure 3:**
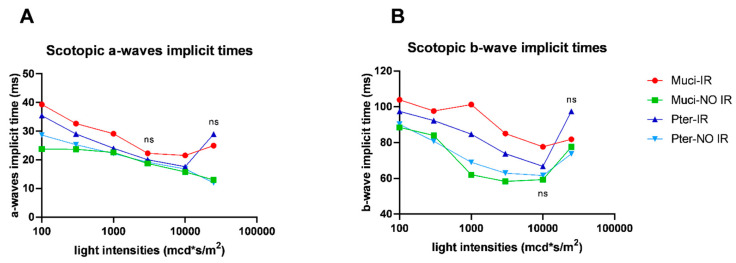
Implicit time analysis of a-wave and b-wave responses in electroretinography (ERG): (**A**) comparison of a-wave implicit times among different treatment groups; (**B**) comparison of b-wave implicit times among different treatment groups. The statistical analysis showed no significant differences in implicit times among the different treatment groups. Data represent mean ± SEM; *p* > 0.05. ns = no significant difference.

The implicit time analysis of a-wave and b-wave responses did not reveal any statistically significant differences among the various treatment groups (Figure 3A,B). 

### 3.2. Western Blot

**Figure 4:**
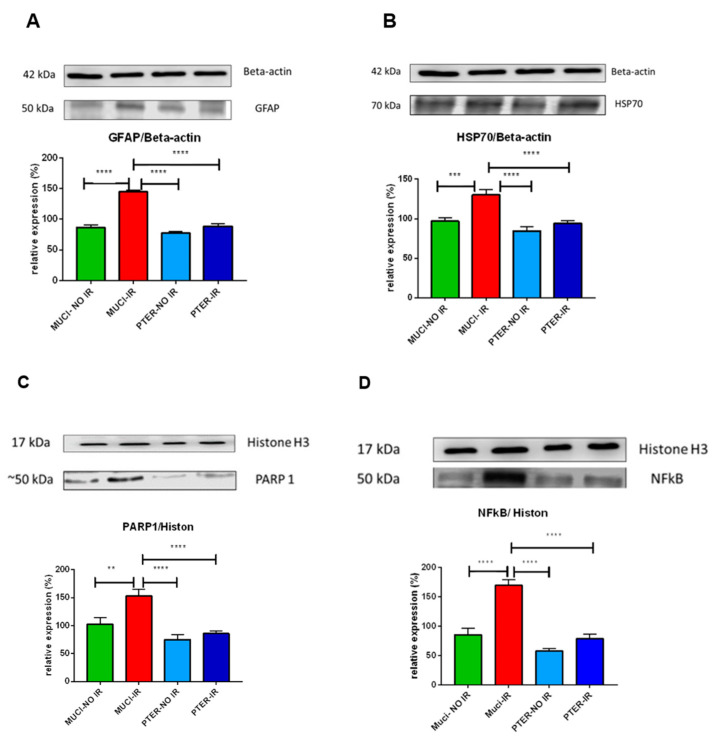
Expression levels of glial fibrillary acidic protein (GFAP) and heat shock protein 70 (HSP70) in retinal tissue: (**A**) Western blot analysis revealing a notable increase in GFAP expression in different groups; (**B**) Western blot analysis demonstrating a similar pattern with elevated levels of HSP70 in different groups. Data represent mean ± SEM; *** *p* < 0.001, **** p < 0.0001, *n* = 4 per group. Expression levels of poly(ADP-ribose) polymerase 1 (PARP1) and nuclear factor kappa B (NFkB) in retinal tissue; (**C**) Western blot analysis showing significantly elevated expression of PARP1 in different groups; (**D**) Western blot analysis revealing a similar pattern with significantly increased levels of NFkB in different groups. Data represent mean ± SEM; ** *p* < 0.01, **** *p* < 0.0001, *n* = 4 per group.

The results of Western blot analysis revealed a notable increase in the expression levels of glial fibrillary acidic protein (GFAP) and heat shock protein 70 (HSP70) in the MUCI IR group compared to both the MUCI NO IR and PTER IR groups. Statistical analysis demonstrated a significant difference when comparing the MUCI NO IR group with these two experimental groups, indicating a distinctive response to I/R injury (Figure 4A,B). Similarly, the expression levels of poly(ADP-ribose) polymerase 1 (PARP1) and nuclear factor kappa B (NFkB) were significantly elevated in the MUCI IR group relative to the MUCI NO IR and PTER IR groups. The observed differences between the groups were statistically significant (Figure 4C,D).

Notably, pterostilbene treatment led to a substantial decrease in the expression levels of all four proteins when compared to animals treated with the vehicle (MUCI), irrespective of whether they were subjected to ischemic reperfusion (MUCI IR) or were in the non-ischemic control group (MUCI NO IR).

### 3.3. Histology

**Figure 5:**
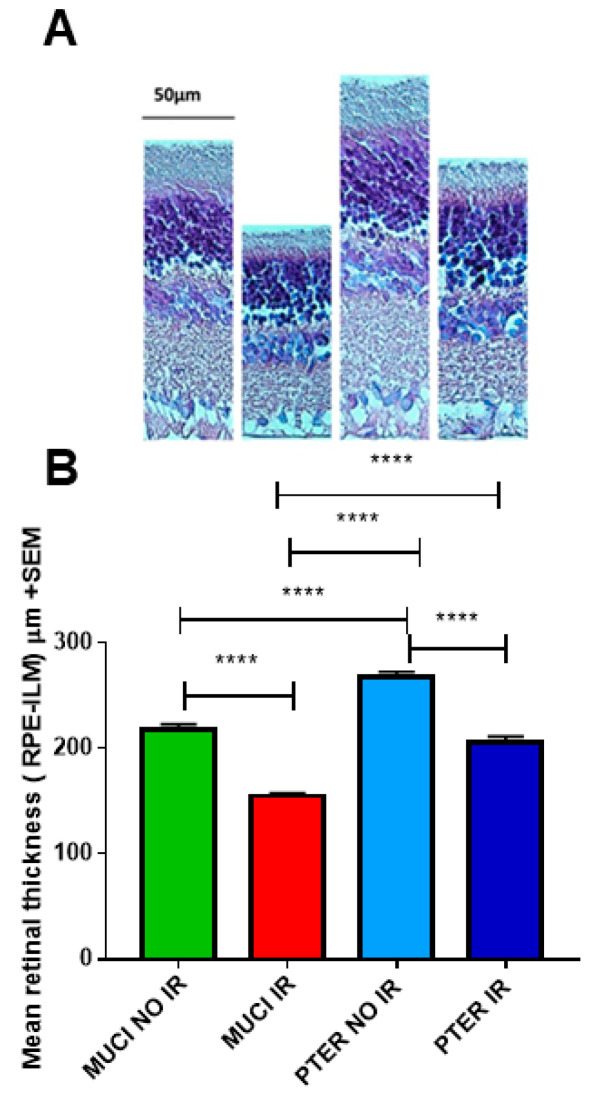
Microscopic analysis of rat retinal sections: (**A**) representative images of retinal thickness in the different treatment groups (from left to right): MUCI NO IR, MUCI IR, PTER NO IR, and PTER IR; (**B**) graphs showing statistical analysis results of histology sections of the different groups. Data represent mean ± SEM; **** *p* < 0.0001.

Based on histological analyses, the following can be reported: Significant differences were observed in the total retinal layer thickness among the four animal groups. This value was found to be the smallest in the MUCI IR group, indicating that the retina thickness in this group was the thinnest compared to the other three animal groups. There was a significant difference between the ischemic (MUCI IR) and the corresponding non-ischemic (MUCI NO IR) animals’ values. The average retinal layer thickness in the latter group was significantly larger, albeit not as much as in the case of non-ischemic animals receiving pterostilbene treatment (PTER NO IR). We compared the retinal thickness of non-ischemic animals in the two treatment groups (MUCI NO IR vs. PTER NO IR). The difference was significant. The same was observed in the case of ischemic eyes (MUCI IR vs. PTER IR). In animals treated with pterostilbene, significant differences in the retinal layer thickness were observed. The values of the PTER IR group were significantly smaller than those of the PTER NO IR group. It can be said that the total retinal thickness was the highest in the PTER NO IR group, considering all four groups (Figure 5A,B).

## 4. Discussion

In our present study, we investigated the effects of pterostilbene on retinal I/R injury in a rat model. Our results demonstrate that pterostilbene has a significant retinoprotective effect, as evidenced by improved retinal function; preserved retinal structure; and reduced markers of glial activation, cellular stress, inflammation, and apoptosis. Pterostilbene treatment significantly enhanced retinal function, as evidenced by the increased b-wave amplitude in ERG recordings. This suggests that pterostilbene preserves the functionality of retinal cells, particularly bipolar and Müller cells, which are crucial for visual signal transmission [36,37]. This indicates that pterostilbene not only protects retinal cells from immediate damage but also helps maintain their functional integrity, which is essential for visual processing. Histological analysis revealed that pterostilbene-treated rats had reduced retinal thinning and better-preserved retinal architecture compared to vehicle-treated rats. This structural preservation is critical because retinal thinning, due to the loss of retinal ganglion cells and inner retinal neurons, commonly results from I/R injury, leading to vision impairment or loss [38,39]. The decreased expression of glial fibrillary acidic protein (GFAP) in pterostilbene-treated rats suggests the mitigation of glial activation, reducing the detrimental effects of gliosis on retinal neurons [40,41]. Gliosis, characterized by the proliferation and hypertrophy of glial cells, can exacerbate neuronal damage and disrupt the retinal environment. By limiting this response, pterostilbene helps maintain a healthier retinal milieu, conducive to neuron survival and function [42]. Furthermore, we found reduced levels of heat shock protein 70 (HSP70), a protein associated with cellular stress responses, indicating that pterostilbene may enhance cellular resilience to I/R-induced stress [43]. The lower expression of HSP70 in pterostilbene-treated rats suggests that these cells experience less stress, possibly due to the antioxidant properties of pterostilbene, which help neutralize the reactive oxygen species (ROS) generated during I/R injury [44,45,46]. The neuroprotective effects of pterostilbene observed in this study are consistent with prior research, which emphasizes its anti-inflammatory, antioxidant, and anti-apoptotic properties [47,48]. Furthermore, pterostilbene has been shown to modulate inflammatory pathways by inhibiting nuclear factor kappa B (NFκB) activation, a key regulator of inflammation [49]. Our findings of decreased NFκB expression in pterostilbene-treated rats corroborate these studies, suggesting that pterostilbene may exert its protective effects partly by reducing inflammatory responses. NFκB upregulates various inflammatory cytokines and adhesion molecules, contributing to secondary damage following I/R injury. By inhibiting this pathway, pterostilbene reduces the inflammatory cascade that exacerbates retinal damage [25,50]. Additionally, the decrease in poly(ADP-ribose) polymerase 1 (PARP1) expression supports the idea that pterostilbene can inhibit apoptosis, as PARP1 is integral to DNA damage-induced cell death pathways. The overactivation of PARP1, in response to significant DNA damage, results in cellular energy depletion and necrotic cell death. By lowering PARP1 expression, pterostilbene likely disrupts this energy-draining cycle, thereby preserving cell viability and function [50,51,52].

Our study enhances the understanding of pterostilbene’s protective effects by offering detailed insights into its impact on retinal I/R injury. While previous research has mainly concentrated on the systemic or neural protective effects of pterostilbene, our work emphasizes its potential as a therapeutic agent for retinal neurodegenerative conditions [44,47,53]. The specific reduction in markers of inflammation, oxidative stress, and apoptosis underscores the multi-faceted protective mechanisms of pterostilbene. Furthermore, our findings suggest that pterostilbene’s protective effects extend to preserving long-term retinal structure and function. This indicates the potential for pterostilbene to be used not only as a treatment to prevent acute damage but also as a long-term therapeutic strategy to maintain retinal health and function. The findings of this study have significant implications for treating retinal I/R injuries and related disorders. By demonstrating that pterostilbene can effectively reduce oxidative stress, inflammation, and apoptosis, this study highlights its potential as a neuroprotective therapeutic agent. These properties are particularly relevant for conditions such as diabetic retinopathy, age-related macular degeneration, glaucoma, and central retinal artery occlusion, where I/R injury is a critical pathogenic mechanism. In diabetic retinopathy, retinal I/R injury contributes to disease progression by promoting inflammation and neuronal damage. Pterostilbene’s ability to mitigate these harmful processes indicates that it could serve as a valuable adjunct therapy for managing diabetic retinopathy [39,54,55,56,57].

This study has several limitations. It was performed on a rat model, so further research is needed to assess its applicability to humans. The dosage and administration method of pterostilbene might require optimization for clinical use, including evaluating its route of administration, bioavailability, and potential side effects. Additionally, this study primarily addressed the acute phase of retinal I/R injury; long-term studies are needed to confirm if pterostilbene’s protective effects are sustained and translate into functional vision improvements. The potential interactions of pterostilbene with other treatments for retinal diseases also need further investigation. 

Obviously, future research should focus on exploring the long-term effects of pterostilbene treatment and its efficacy in different models of retinal injury. Additionally, understanding the precise molecular mechanisms by which pterostilbene exerts its protective effects will be crucial for developing targeted therapies. Clinical trials will also be necessary to confirm the safety and effectiveness of pterostilbene in human subjects. Another avenue for future research is to investigate the potential synergistic effects of pterostilbene with other neuroprotective agents. Combining pterostilbene with established treatments for retinal diseases could enhance therapeutic outcomes and provide more comprehensive protection against I/R injury. 

In conclusion, our research suggests that pterostilbene, as a potential future adjunct treatment, may reduce retinal injury caused by I/R and enhance functional retinal status. By reducing oxidative stress, inflammation, and apoptosis, pterostilbene could improve visual acuity. Additionally, since numerous clinical studies have linked visual loss with cognitive decline, pterostilbene may also contribute to an improved quality of life [58,59].

## Figures and Tables

**Table 1:** Experimental structure.

Groups	Number of Animals	Treatments	Examinations
1	10 male SD rats	Pterostilbene-treated group = Left ligated eyes = pterostilbene-treated ischemic reperfusion group (PTER IR) Right non-ischemic eyes = the pterostilbene-treated non-ischemic group (PTER NO IR)/	Electroretinography, Western blot, and histology
2	10 male SD rats	Vehicle (Mucilago)-treated group = Left ligated eyes = vehicle-treated ischemic–reperfusion group (MUCI IR) Right non-ischemic eyes = the vehicle-treated non-ischemic control (MUCI NO IR)	

## Data Availability

Datasets are available upon request from the authors; however, restrictions may apply as the datasets presented in this article are the property of the University of Debrecen. Requests to access the datasets should be directed to the authors.

## References

[1] Chinskey N.D., Besirli C.G., Zacks D.N. (2014). Retinal cell death and current strategies in retinal neuroprotection. Curr. Opin. Ophthalmol..

[2] Osborne N.N., Casson R.J., Wood J.P., Chidlow G., Graham M., Melena J. (2004). Retinal ischemia: Mechanisms of damage and potential therapeutic strategies. Prog. Retin. Eye Res..

[3] Bresnick G.H., De Venecia G., Myers F.L., Harris J.A., Davis M.D. (1975). Retinal ischemia in diabetic retinopathy. Arch. Ophthalmol..

[4] Wu D., An Q., Ji H., Dai J., Suo L., Zhang C. (2023). Retinal ischemia-reperfusion injury induces intense lipid synthesis and remodeling. Biochem. Biophys. Res. Commun..

[5] Qin Q., Yu N., Gu Y., Ke W., Zhang Q., Liu X., Wang K., Chen M. (2022). Inhibiting multiple forms of cell death optimizes ganglion cells survival after retinal ischemia reperfusion injury. Cell Death Dis..

[6] Jurcau A., Ardelean I.A. (2021). Molecular pathophysiological mechanisms of ischemia/reperfusion injuries after recanalization therapy for acute ischemic stroke. J. Integr. Neurosci..

[7] Li R., Jia Z., Trush M.A. (2016). Defining ROS in Biology and Medicine. React. Oxyg. Species.

[8] Chandrasekaran A., Idelchik M., Melendez J.A. (2017). Redox control of senescence and age-related disease. Redox Biol..

[9] Islam M.T. (2017). Oxidative stress and mitochondrial dysfunction-linked neurodegenerative disorders. Neurol. Res..

[10] Carrera-Juliá S., Moreno M.L., Barrios C., de la Rubia Ortí J.E., Drehmer E. (2020). Antioxidant Alternatives in the Treatment of Amyotrophic Lateral Sclerosis: A Comprehensive Review. Front. Physiol..

[11] Abtahi S.H., Nourinia R., Mazloumi M., Nouri H., Arevalo J.F., Ahmadieh H. (2023). Retinal ischemic cascade: New insights into the pathophysiology and imaging findings. Surv. Ophthalmol..

[12] Rosenzweig R., Nillegoda N.B., Mayer M.P., Bukau B. (2019). The Hsp70 chaperone network. Nat. Rev. Mol. Cell Biol..

[13] Kim J.Y., Han Y., Lee J.E., Yenari M.A. (2018). The 70-kDa heat shock protein (Hsp70) as a therapeutic target for stroke. Expert. Opin. Ther. Targets.

[14] Jurga A.M., Paleczna M., Kadluczka J., Kuter K.Z. (2021). Beyond the GFAP-Astrocyte Protein Markers in the Brain. Biomolecules.

[15] Greenwald S.H., Pierce E.A. (2019). Parthanatos as a Cell Death Pathway Underlying Retinal Disease. Adv. Exp. Med. Biol..

[16] Lawrence T. (2009). The nuclear factor NF-kappaB pathway in inflammation. Cold Spring Harb. Perspect. Biol..

[17] King R.E., Kent K.D., Bomser J.A. (2005). Resveratrol reduces oxidation and proliferation of human retinal pigment epithelial cells via extracellular signal-regulated kinase inhibition. Chem. Biol. Interact..

[18] He Q., Xiao L., Shi Y., Li W., Xin X. (2023). Natural products: Protective effects against ischemia-induced retinal injury. Front. Pharmacol..

[19] Li L., Wang Y., Qin X., Zhang J., Zhang Z. (2018). Echinacoside protects retinal ganglion cells from ischemia/reperfusion-induced injury in the rat retina. Mol. Vis..

[20] Silfen J., Yanai P., Cabantchik Z.I. (1988). Bioflavonoid effects on in vitro cultures of Plasmodium falciparum. Inhibition of permeation pathways induced in the host cell membrane by the intraerythrocytic parasite. Biochem. Pharmacol..

[21] Roupe K.A., Remsberg C.M., Yáñez J.A., Davies N.M. (2006). Pharmacometrics of stilbenes: Seguing towards the clinic. Curr. Clin. Pharmacol..

[22] Tsai H.Y., Ho C.T., Chen Y.K. (2017). Biological actions and molecular effects of resveratrol, pterostilbene, and 3'-hydroxypterostilbene. J. Food Drug Anal..

[23] Huang W., Yan Z., Li D., Ma Y., Zhou J., Sui Z. (2018). Antioxidant and Anti-Inflammatory Effects of Blueberry Anthocyanins on High Glucose-Induced Human Retinal Capillary Endothelial Cells. Oxid. Med. Cell Longev..

[24] Li J., Ruzhi D., Hua X., Zhang L., Lu F., Coursey T.G., Pflugfelder S.C., Li D.Q. (2016). Blueberry Component Pterostilbene Protects Corneal Epithelial Cells from Inflammation via Anti-oxidative Pathway. Sci. Rep..

[25] Surien O., Masre S.F., Basri D.F., Ghazali A.R. (2023). Potential Chemopreventive Role of Pterostilbene in Its Modulation of the Apoptosis Pathway. Int. J. Mol. Sci..

[26] Rimando A.M., Kalt W., Magee J.B., Dewey J., Ballington J.R. (2004). Resveratrol, pterostilbene, and piceatannol in vaccinium berries. J. Agric. Food Chem..

[27] Xue E.X., Lin J.P., Zhang Y., Sheng S.R., Liu H.X., Zhou Y.L., Xu H. (2017). Pterostilbene inhibits inflammation and ROS production in chondrocytes by activating Nrf2 pathway. Oncotarget.

[28] de Queiroz K.B., Dos Santos Fontes Pereira T., Araújo M.S.S., Gomez R.S., Coimbra R.S. (2018). Resveratrol Acts Anti-Inflammatory and Neuroprotective in an Infant Rat Model of Pneumococcal Meningitis by Modulating the Hippocampal miRNome. Mol. Neurobiol..

[29] Paul S., Rimando A.M., Lee H.J., Ji Y., Reddy B.S., Suh N. (2009). Anti-inflammatory action of pterostilbene is mediated through the p38 mitogen-activated protein kinase pathway in colon cancer cells. Cancer Prev. Res..

[30] Wang B., Liu H., Yue L., Li X., Zhao L., Yang X., Wang X., Yang Y., Qu Y. (2016). Neuroprotective effects of pterostilbene against oxidative stress injury: Involvement of nuclear factor erythroid 2-related factor 2 pathway. Brain Res..

[31] Liu H., Wu X., Luo J., Wang X., Guo H., Feng D., Zhao L., Bai H., Song M., Liu X. (2019). Pterostilbene Attenuates Astrocytic Inflammation and Neuronal Oxidative Injury After Ischemia-Reperfusion by Inhibiting NF-κB Phosphorylation. Front. Immunol..

[32] Qu X., Zhang L., Wang L. (2023). Pterostilbene as a Therapeutic Alternative for Central Nervous System Disorders: A Review of the Current Status and Perspectives. J. Agric. Food Chem..

[33] Nagarajan S., Mohandas S., Ganesan K., Xu B., Ramkumar K.M. (2022). New Insights into Dietary Pterostilbene: Sources, Metabolism, and Health Promotion Effects. Molecules.

[34] Szilágyi A., Takács B., Szekeres R., Tarjányi V., Bombicz M., Priksz D., Kovács A., Juhász B., Frecska E., Szilvássy Z. (2022). Therapeutic Properties of Ayahuasca Components in Ischemia/Reperfusion Injury of the Eye. Biomedicines.

[35] Varga B., Gesztelyi R., Bombicz M., Haines D., Szabo A.M., Kemeny-Beke A., Antal M., Vecsernyes M., Juhasz B., Tosaki A. (2013). Protective effect of alpha-melanocyte-stimulating hormone (α-MSH) on the recovery of ischemia/reperfusion (I/R)-induced retinal damage in a rat model. J. Mol. Neurosci..

[36] Ozawa Y., Toda E., Kawashima H., Homma K., Osada H., Nagai N., Abe Y., Yasui M., Tsubota K. (2019). Aquaporin 4 Suppresses Neural Hyperactivity and Synaptic Fatigue and Fine-Tunes Neurotransmission to Regulate Visual Function in the Mouse Retina. Mol. Neurobiol..

[37] Lawrenson J.G., Hull C.C., Downie L.E. (2017). The effect of blue-light blocking spectacle lenses on visual performance, macular health and the sleep-wake cycle: A systematic review of the literature. Ophthalmic Physiol. Opt..

[38] Luo H., Zhuang J., Hu P., Ye W., Chen S., Pang Y., Li N., Deng C., Zhang X. (2018). Resveratrol Delays Retinal Ganglion Cell Loss and Attenuates Gliosis-Related Inflammation From Ischemia-Reperfusion Injury. Investig. Ophthalmol. Vis. Sci..

[39] Chronopoulos P., Manicam C., Zadeh J.K., Laspas P., Unkrig J.C., Göbel M.L., Musayeva A., Pfeiffer N., Oelze M., Daiber A. (2023). Effects of Resveratrol on Vascular Function in Retinal Ischemia-Reperfusion Injury. Antioxidants.

[40] Fang X.L., Zhang Q., Xue W.W., Tao J.H., Zou H.D., Lin Q.R., Wang Y.L. (2023). Suppression of cAMP/PKA/CREB signaling ameliorates retinal injury in diabetic retinopathy. Kaohsiung J. Med. Sci..

[41] Subirada P.V., Vaglienti M.V., Joray M.B., Paz M.C., Barcelona P.F., Sánchez M.C. (2022). Rapamycin and Resveratrol Modulate the Gliotic and Pro-Angiogenic Response in Müller Glial Cells Under Hypoxia. Front. Cell Dev. Biol..

[42] Guo J., Wang J., Guo R., Shao H., Guo L. (2022). Pterostilbene Protects the Optic Nerves and Retina in a Murine Model of Experimental Autoimmune Encephalomyelitis via Activation of SIRT1 Signaling. Neuroscience.

[43] Liu J., Fan C., Yu L., Yang Y., Jiang S., Ma Z., Hu W., Li T., Yang Z., Tian T. (2016). Pterostilbene exerts an anti-inflammatory effect via regulating endoplasmic reticulum stress in endothelial cells. Cytokine.

[44] Li Y.R., Li S., Lin C.C. (2018). Effect of resveratrol and pterostilbene on aging and longevity. Biofactors.

[45] Shen H., Rong H. (2015). Pterostilbene impact on retinal endothelial cells under high glucose environment. Int. J. Clin. Exp. Pathol..

[46] Lee D., Fu Z., Hellstrom A., Smith L.E.H. (2024). Therapeutic Effects of Anti-Inflammatory and Anti-Oxidant Nutritional Supplementation in Retinal Ischemic Diseases. Int. J. Mol. Sci..

[47] Remsberg C.M., Yáñez J.A., Ohgami Y., Vega-Villa K.R., Rimando A.M., Davies N.M. (2008). Pharmacometrics of pterostilbene: Preclinical pharmacokinetics and metabolism, anticancer, antiinflammatory, antioxidant and analgesic activity. Phytother. Res..

[48] Dvorakova M., Landa P. (2017). Anti-inflammatory activity of natural stilbenoids: A review. Pharmacol. Res..

[49] Lin Y.J., Ding Y., Wu J., Ning B.T. (2016). Pterostilbene as treatment for severe acute pancreatitis. Genet. Mol. Res..

[50] Li J., Xie C., Zhuang J., Li H., Yao Y., Shao C., Wang H. (2015). Resveratrol attenuates inflammation in the rat heart subjected to ischemia-reperfusion: Role of the TLR4/NF-κB signaling pathway. Mol. Med. Rep..

[51] Wang Y., Luo W., Wang Y. (2019). PARP-1 and its associated nucleases in DNA damage response. DNA Repair.

[52] Liu S., Luo W., Wang Y. (2022). Emerging role of PARP-1 and PARthanatos in ischemic stroke. J. Neurochem..

[53] Kim H., Seo K.H., Yokoyama W. (2020). Chemistry of Pterostilbene and Its Metabolic Effects. J. Agric. Food Chem..

[54] Zhang K., Wang T., Sun G.F., Xiao J.X., Jiang L.P., Tou F.F., Qu X.H., Han X.J. (2023). Metformin protects against retinal ischemia/reperfusion injury through AMPK-mediated mitochondrial fusion. Free Radic. Biol. Med..

[55] Zheng L., Gong B., Hatala D.A., Kern T.S. (2007). Retinal ischemia and reperfusion causes capillary degeneration: Similarities to diabetes. Investig. Ophthalmol. Vis. Sci..

[56] Gange W.S., Qiao J.B., Park P.J., McDonnell J.F., Tan Z., Perlman J.I., Bu P. (2021). Protection of Retinal Function by Nucleoside Reverse Transcriptase Inhibitors Following Retinal Ischemia/Reperfusion Injury. J. Ocul. Pharmacol. Ther..

[57] Renner M., Stute G., Alzureiqi M., Reinhard J., Wiemann S., Schmid H., Faissner A., Dick H.B., Joachim S.C. (2017). Optic Nerve Degeneration after Retinal Ischemia/Reperfusion in a Rodent Model. Front. Cell Neurosci..

[58] Kwan R.Y.C., Kwan C.W., Kor P.P.K., Chi I. (2022). Cognitive decline, sensory impairment, and the use of audio-visual aids by long-term care facility residents. BMC Geriatr..

[59] Aubin G., Phillips N., Jaiswal A., Johnson A.P., Joubert S., Bachir V., Kehayia E., Wittich W. (2023). Visual and cognitive functioning among older adults with low vision before vision rehabilitation: A pilot study. Front. Psychol..

